# Infectious Mononucleosis Revealed by Non-steroidal Anti-inflammatory Drug: A First Clinical Report

**DOI:** 10.7759/cureus.60329

**Published:** 2024-05-15

**Authors:** Ashraf I Ahmed, Hamad A Alkorbi, Lolwa Jolo, Maha Al Kurbi, Shahem Abbarh, Mohammed Danjuma

**Affiliations:** 1 Internal Medicine, College of Medicine, QU Health, Qatar University, Doha, QAT; 2 Internal Medicine, Hamad Medical Corporation, Doha, QAT; 3 College of Medicine and Surgery, Almaarefa University, Riyadh, SAU

**Keywords:** non-steroidal anti-inflammatory drugs (nsaids), erythema, rash, epstein-barr virus (ebv), infectious mononucleosis (im)

## Abstract

Infectious mononucleosis (IM), primarily caused by the Epstein-Barr virus (EBV), is a common viral illness among adolescents and young adults. IM typically presents with symptoms such as fever, lymphadenopathy, and pharyngitis. We present a case of a 32-year-old woman who developed a maculopapular rash following ibuprofen administration, revealing an underlying undiagnosed IM. Laboratory investigations confirmed EBV infection. This represents the first documented case linking non-steroidal anti-inflammatory drugs (NSAIDs) to IM presentation. Awareness of this association is crucial for timely diagnosis and management, especially when evaluating patients with unexplained skin reactions to medications.

## Introduction

Infectious mononucleosis (IM), primarily caused by the Epstein-Barr virus (EBV), is one of the most prevalent viral morbidities among adolescents and young adults aged 15-24 years [1]. It is primarily transmitted via oral shedding in salivary secretions. EBV can also spread during sexual contact through bodily fluids and blood [2]. IM typically manifests as fever, sore throat, and generalized lymphadenopathy [3]. In addition, phenotypic variation, such as diffuse maculopapular skin rash, may also be seen in some IM cases. Although a unifying trigger for all cases differs, antibiotic exposure, particularly to penicillins (such as ampicillin and amoxicillin), has frequently been linked with it [3]. The primary mechanism for skin rash development in IM appears to be a transient virus-mediated immune alteration, potentially leading to a reversible hypersensitivity reaction [4]. Although some studies suggest that IM patients have persistent medication allergies, it's not clear if this is a distinct phenomenon contributing to the overall pathogenesis of the disease [4].

While most cases of IM are self-limiting, morbidities associated with it sometimes necessitate symptomatic therapy for pain relief and the management of other potential complications. The most common among these include bed rest, adequate hydration, and the use of over-the-counter painkillers, such as non-steroidal anti-inflammatory drugs (NSAIDs) [5]. Although the use of NSAIDs in IM for symptomatic relief is generally considered safe, a few reports have suggested that the transient virus-mediated immune alteration extends beyond the antibiotics and might be observed in other medications [4]. Hence, in this case report, we describe the first case of a young female who presented with an NSAID-induced rash revealing an underlying infectious mononucleosis. This highlights the importance for primary healthcare providers to exercise caution when prescribing medications to patients with persistent fever and remain vigilant for signs of adverse drug reactions (ADR). Furthermore, what might seem as just an ADR to NSAID might actually be a developing IM.

## Case presentation

A previously healthy 32-year-old Qatari woman presented to the ED with a sudden onset of skin rash. The patient reported persistent fever (40°C), sore throat, and mild abdominal pain for 10 days. On the day of the presentation, she self-medicated with ibuprofen 400 mg. A few hours after taking the ibuprofen tablet, the patient developed a new-onset, non-pruritic diffuse skin rash across her entire body (Naranjo score of 5). She denied the use of any other drug or new herbs. Her past medical history was unremarkable. There were no previous known allergies. She is married and sexually active in a monogamous relationship with her husband. She denied a history of smoking or alcohol consumption.

On examination, vital signs were stable, apart from high-grade fever and a heart rate of 110 beats per minute. Chest and cardiovascular examinations were unremarkable. The abdomen was soft and non-tender, with no hepatosplenomegaly. There was a systemic rash characterized by a small, red, flat, discolored area and raised bumps that tended to merge into more prominent papules, particularly predominant on the arms and both thighs (The patient did not consent to obtain images of the skin rash for cultural and religious beliefs). Laboratory investigations revealed a high level of C-reactive protein (24.4 mg/L), white blood cell count (WBC) was 5.4 x 10^3^/uL, with absolute neutrophil count of 1.5 x 10^3^/uL, lymphocyte count of 3.8 x 10^3^/uL, and normal eosinophil count. Peripheral smear revealed many reactive "atypical" lymphocytes. There were also elevated liver enzyme levels [Aspartate Transaminase (AST): 100 U/L, Alanine Transaminase (ALT): 184 U/L, Alkaline Phosphatase: 131 U/L)]. Urinalysis revealed microcytic hematuria (red blood cell count of 82 cells/ul), a WBC count of 43 cells/uL, and proteinuria 2+. A urine culture later showed no growth. Serological testing confirmed the presence of EBV antibodies IgM. Virology and serology were negative against human immunodeficiency virus, influenzas A/B, respiratory syncytial viruses, and Covid-19.

The patient was admitted for observation for a day in the hospital. She was assessed by the dermatology team and was offered a skin biopsy, which she refused. Subsequently, a clinical diagnosis of IM was highly suggested. The patient received symptomatic management, including intravenous hydration and, as needed, paracetamol for fever. The rash started to fade the next day, and she was discharged. A follow-up appointment after one week revealed complete resolution of the rash and fever with improved liver enzyme levels.

## Discussion

This case report highlights an adult female with a history of persistent fever who subsequently developed a maculopapular rash after the initiation of NSAIDs, revealing a most likely underlying undiagnosed IM. To our knowledge, this represents the first report of an adjudicated causality between NSAID exposure and presentation with phenotypic and laboratory features consistent with IM. In contrast, other reports were found to document cases of IM with maculopapular exanthem after intake of beta-lactam antibiotics, such as ampicillin and amoxicillin [4]. For example, Ciccarese et al. reported that ampicillin and amoxicillin were the most frequently reported medications to cause maculopapular exanthem in patients with IM caused by EBV [6]. Moreover, Baciewicz and Chandra reported a case of an 18-year-old female with IM who developed a pruritic maculopapular rash after the use of cefprozil [7]. Similarly, a 23-year-old male developed a diffuse maculopapular rash two days after the initiation of azithromycin (Naranjo score of 6) [8].

Other reports, such as those by Ónodi-Nagy et al., have raised the need for increased awareness and vigilance by physicians and other healthcare providers on the need to be more open to the possibility of more links between skin rash cases with other drugs [4]. The underlying mechanism that underpins the immunological reaction remains unclear. It has been suggested that EBV infection is principally associated with transient immunomodulation, leading to delayed-type drug hypersensitivity [4]. To illustrate, the viral infection results in CD8+ T-lymphocytosis, releasing a pool of cytokines, including interferon-gamma, interleukin-2, and TNF-alpha. This derives the CD4+ lymphocyte differentiation toward type 1 helper t-lymphocytes (TH1) lineage. Simultaneously, it deprives the production of Th2-mediated cytokines Interleukin 5, 6, and, most importantly, Interleukin-10, which leads to loss of antigenic tolerance and hypersensitivity [4]. On the other hand, most xenobiotics, including arylpropionic acids, are intrinsically immunogenic but do not lead to cell-mediated immunity activation due to their relatively small size [9]. However, Nanau and Neuman proposed that during viral hepatitis, an enhanced major histocompatibility complex presentation is observed for liver metabolites, increasing the likelihood of immunological recognition and reaction [9]. Additionally, the transient immunomodulation induced by the viral infection represents the cornerstone of the drug-induced skin eruption. Nevertheless, the induction of a lasting drug-specific sensitization remains unknown. Ciccarese et al. reported that future adverse reactions to the same drugs are unpredictable [6]. Hence, it is favored to implement conventional skin tests to anticipate subsequent adverse reactions.

That is, the case presented here highlights the importance of considering IM as a potential underlying cause of medication-induced skin reactions in the proper clinical settings. This is secondary to the immune modulation, which sets a lower threshold for skin eruption even for medications that were not known to induce adverse skin reactions. This further supports the importance of primary healthcare providers being circumspect in evaluating patients with persistent fever and remaining vigilant for possible features of adverse drug reactions, which may be harbingers of IM.

## Conclusions

This case underscores the potential association between the administration of NSAIDs and the manifestation of infectious mononucleosis (IM). While previous literature has primarily linked IM presentation to antibiotics like ampicillin and amoxicillin, our report highlights the need for physicians to remain vigilant when encountering patients on NSAID therapy who develop unexplained maculopapular rash. This suggests a broader range of medications that may be implicated in triggering IM-related skin reactions. It emphasizes the importance of considering IM as a possible underlying cause of medication-induced skin eruptions, especially in patients presenting with persistent fever. This insight can guide primary healthcare providers in the timely recognition and appropriate management of adverse drug reactions associated with IM.
